# HPV and Cytology Testing in Women Undergoing 9-Valent HPV Opportunistic Vaccination: A Single-Cohort Follow Up Study

**DOI:** 10.3390/vaccines9060643

**Published:** 2021-06-12

**Authors:** Rosa De Vincenzo, Nicola Caporale, Valentina Bertoldo, Caterina Ricci, Maria Teresa Evangelista, Nicolò Bizzarri, Luigi Pedone Anchora, Giovanni Scambia, Giovanni Capelli

**Affiliations:** 1 Gynecologic Oncology Unit, Department of Woman and Child Health and Public Health, Fondazione Policlinico Universitario A. Gemelli, IRCCS, 00168 Roma, Italy; nicola.caporale01@icatt.it (N.C.); valentina.bertoldo@guest.policlinicogemelli.it (V.B.); caterina.ricci@policlinicogemelli.it (C.R.); mariateresa.evangelista@policlinicogemelli.it (M.T.E.); nicolo.bizzarri@policlinicogemelli.it (N.B.); luigi.pedoneanchora@policlinicogemelli.it (L.P.A.); giovanni.scambia@policlinicogemelli.it (G.S.); 2 Dipartimento di Scienze della Vita e Sanità Pubblica, Università Cattolica del Sacro Cuore, 00168 Roma, Italy; 3 Dipartimento di Scienze Umane, Sociali e della Salute, Università di Cassino e del Lazio Meridionale, 03043 Cassino, Italy

**Keywords:** human papillomavirus (HPV), 9vHPV vaccine, adult women, Pap smear, HPV DNA test, HPV clearance

## Abstract

**Background**: This study evaluates the possible effect of 9-valent (9vHPV) vaccination on the results of HPV and cytological tests in a cohort of adult women. **Methods**: This study is a retrospective, single-cohort, monocentric study. Sexually active women aged 14–70 years, who underwent 9vHPV vaccination, were enrolled. Dose administration dates, side effects and data on Pap smears and HPV tests performed before and after the first vaccine dose were collected. Subjects were considered “unexposed” to the vaccine for all time intervals before the first dose administration, and “exposed” to the first, second and third vaccine doses in all time intervals following each specific dose. **Results**: A total of 512 women underwent the first 9vHPV dose administration and were enrolled in the study. Median age at vaccination was 30.5 (14–70). Log-rank tests and Cox regression analyses showed a highly statistically significant (*p* < 0.0001) difference in the time to negativization after the exposure to the third vaccine dose in the 207 women starting with a Pap+ smear (HR (95% C.I.), 2.66 (1.83–3.86)) and in the 198 women starting with an HPV HR+ test (HR (95% C.I.), 7.80 (4.83–12.60)). **Conclusions**: 9vHPV vaccination may play a role in shortening the clearance time of HPV HR+ or Pap positivity in sexually active adult women.

## 1. Introduction

Human papillomavirus (HPV) is the most common sexually transmitted infection of the lower genital tract worldwide, with an estimated lifetime risk of infection greater than 80%. Persistent infection with high-risk HPV types is associated with precancerous and cancerous lesions [1,2]. Prophylactic HPV vaccination is a powerful strategy aimed to reduce and possibly eradicate HPV-related disease and cervical cancer [3]. In clinical trials and in long-term follow-up studies, HPV vaccines showed impressive immunogenicity, efficacy and safety, especially in naïve populations [4,5,6]. 

The World Health Organization (WHO) still recognizes HPV-related disease as a major global public health issue and recommends HPV vaccination in all national public programs [7,8]. Since 2006, administration guidelines for vaccines underwent multiple updates regarding age, dosing schedule and gender. In Europe, HPV vaccines have been available since 2006, licensed at the beginning for young females [9]. Few years after, immunological and clinical studies added new important perspectives for adult women vaccination [10,11,12]. Moreover, since 2011, HPV quadrivalent vaccine indication has been extended to boys [13], leading to gender-neutral vaccination programs for children and teenagers, with a catch-up program for young adults. Three different vaccines have been developed thus far [5]: the bivalent vaccine targets HPV types 16/18; the quadrivalent vaccine targets HPV types 6, 11, 16 and 18; and, finally, the 9-valent (9vHPV) vaccine [14] which adds to the same HPV types of the quadrivalent vaccine high-risk types 31, 33, 45, 52 and 58. Regardless of the vaccine, the WHO currently endorses a two-dose series (0–6 months) for boys and girls aged 9–14 and a three-dose schedule (0, 1–2, 6 months) for those aged 15 years or older [7]. 

Different approaches for population and individual HPV vaccination programs have been chosen according to the specificities of national/regional health care systems [9,15].

In Italy, the national HPV vaccination program was implemented in 2007 as an active and free campaign for girls in their 12th year of life, even if some regions opted to extend the free of charge offer to further cohorts, such as girls aged 15 or 16 years or girls aged 25 years, linking it to the first call for screening. Moreover, women aged 14–26 years were given the opportunity of a catch-up vaccination with copayment, in local health unit (ASL) or hospital-based outpatient services. After the introduction of the 2017–2019 National Preventive Vaccination Plan (2017–2019 PNPV) [16], access to vaccination became active and free for all 12-year-old boys and adolescents, with the aim of universal HPV vaccination. Finally, since 2019, the Italian “Calendario Vaccinale della Vita” [17], adopted by general practitioners and some scientific societies, has suggested the possibility of an “opportunistic” vaccination in adult subjects as a personal preventive tool, without age limits [18]. 

In October 2018, the Food and Drug Administration (FDA) approved the extension of the eligibility range for the new 9vHPV vaccine to both women and men aged 27–45 years [19]. In June 2019, the Advisory Committee on Immunization Practice (ACIP) recommended to individuals aged 27–45 years, not yet vaccinated, to discuss the option with their physician [20]. In Europe, the EMA approved the use of the 9vHPV vaccine for active immunization of individuals from the age of 9 years [21], and vaccination of adult populations is not recommended as a priority, too, but it can still be proposed provided that it will not compete for resources to be devoted to the primary targets of vaccination campaigns and cervical cancer screening programs [22,23].

Obviously, the optimal time for HPV immunization lies before to the individual’s sexual debut [24], but HPV vaccination may now be considered as a personal preventive tool in the adult population. Indeed, the vaccine is still safe, well-tolerated and immunogenic at this age [11,12,25]. Moreover, recent studies [26,27] suggested that adjuvant HPV vaccination in patients previously treated for HPV-related disease can reduce disease recurrence. 

Data on the natural history of HPV infection in older women are still controversial. In fact, while the majority of HPV infections are transient and clear within a couple of years following exposure, especially in the young population [28], 10–20% of infections persist latently, leading to disease progression and, ultimately, to various forms of invasive cancer.

The likelihood that a newly detected HPV is due to a new infection versus re-detection of a prior infection (latent HPV infection) declines with age [29]. Moreover, the HPV prevalence generally declines with age, but, in some parts of the world, as reported in Italy [30], a second peak of infection (the U-shaped age-specific prevalence curves) may be observed in middle-aged women [31,32].

However, rates of progression from infection to high-grade lesions are similar in young and adult women [33], and this suggests that also infections acquired during the adult years can lead to high-grade lesions. Moreover, the persistence of oncogenic and non-oncogenic HPV types increases steadily with age [34].

Although HPV vaccines are, nowadays, still not considered to play a therapeutic role in patients with prevalent HPV infection or disease and vaccine efficacy decreases with the increase in age at vaccination [2,35], a possible outcome of interest for vaccination in adult HPV-positive women can be the evolution of the results of diagnostics tests commonly used for monitoring HPV infection (Pap smear and HPV DNA test results) before and after the vaccination. 

This observational retrospective study aims to evaluate the possible effect of the vaccination timing on the results of microbiological and cytological tests in a cohort of adult sexually active women who underwent opportunistic 9vHPV vaccination and to evaluate the potential of the 9vHPV vaccine in boosting HPV viral clearance.

## 2. Materials and Methods

Study design—This is a retrospective, single-cohort, monocentric study. This study was approved by the local ethics committee (protocol ID 3411, 7 August 2020). 

Participants—From our institutional data repositories, we identified sexually active women, aged 14–70 years, who underwent 9vHPV vaccination, between June 2017 and November 2020, in the “PreGIO” gynecologic outpatient clinic, Fondazione Policlinico Universitario Agostino Gemelli, IRCCS, in Rome, Italy. In “PreGIO”, women are vaccinated opportunistically, encouraged by their care provider or by self-decision. Immuno-compromised women (e.g., HIV+) and women with a major abnormal cytological alteration (H-SIL) prior to 9vHPV vaccination are excluded from vaccination. The vaccine used in our outpatient clinic is the 9vHPV vaccine, offered with copayment (at a price for each dose of € 110.00), administered according to the schedule of 0, 2 and 6 months, with an observation of 15 min after dose administrations. 

Data collected—For each patient enrolled in this study, clinical data including age, dose administration dates, side effects and previous HPV-related treatments were collected from the PreGIO archive, and data on Pap smears and HPV tests performed before and after the first vaccine dose were retrieved by the Gemelli Hospital Information System, or by collecting personal exams brought by the women (during vaccination or follow-up visits).

Both conventional Pap smears and ThinPrep samples were considered for the analysis as long as they were reported according to the Bethesda system. Therefore, Pap smear results were reported as negative when negative to intraepithelial lesion or malignancy, or positive if minor abnormal cytological alterations—diagnosis of atypical squamous cells of undetermined significance (ASC-US) or low-grade lesion (LSIL)—were identified. 

Concerning HPV DNA tests, they were executed following different techniques (Digene HC2 or HPV DNA genotyping tests). Digene HC2 is able to differentiate only between 2 HPV DNA groups, low-risk (LR) and high-risk (HR) types. Where genotyping was performed, we defined HR+, LR+ or negative status according to the Anyplex™ II HPV28 Detection Seegene test—performed in Gemelli Hospital—in which HR types are 16, 18, 26, 31, 33, 35, 39, 45, 51, 52, 53, 56, 58, 59, 66, 68, 69, 73, 82; and LR types are 6, 11, 40, 42, 43, 44, 54, 61, 70. 

With the aim to include also the Digene HC2 results in our analysis, we decided to group the HPV results of the genotyping test as HPV HR+ when at least one HR genotype was identified, HPV LR+ when only LR genotypes were identified and HPV-negative when no HPV type was reported. Patients with an HR-HPV test and LSIL were histologically confirmed to be CIN1 or less after colposcopy-guided biopsy.

Statistical analysis—For descriptive analysis of subject characteristics and vaccination, Pap smear and HPV test timings and results, medians, IQRs and range values were calculated and presented as single numbers or visualized by box plots. Inferential analysis was focused on the time to negativization of any positive Pap test or HPV HR+ test, at any time before or after vaccine dose administration. 

In particular, for the analysis of the time to negativization of Pap smears, only women for whom at least 1 positive Pap smear followed by at least 1 other Pap smear (regardless of the result) was retrieved were included in the analysis. On the other hand, for the analysis of the time to negativization of HPV tests, only women for whom at least 1 HPV HR+ test followed by at least 1 other HPV test (regardless of the result) was retrieved were included in the analysis. In this case, HPV LR+ test results were considered as negativizations of a previous HPV HR+ test, such as HPV-negative tests. Exposure to vaccine was considered as a time-changing variable, and subjects were considered “unexposed” for all time intervals before the first dose administration, and “exposed” for the first, second and third vaccine doses in all time intervals following each specific dose. Data on Pap and HPV tests were evaluated following the LOCF (last observation carried forward) criterium for the time intervals among doses, when no further testing was performed in those time intervals. 

The resulting data were analyzed by survival analysis (time-to-event) methods: Kaplan–Meier, log-rank test and Cox regression for the evaluation of potential confounding factors (age in days at event and conization within 1 y before the vaccination 1st dose). All statistical analyses and graphical data displays were performed by Stata 16.1 MP statistical software [36,37]. 

## 3. Results

### 3.1. Sample Characteristics

**Figure 1:**
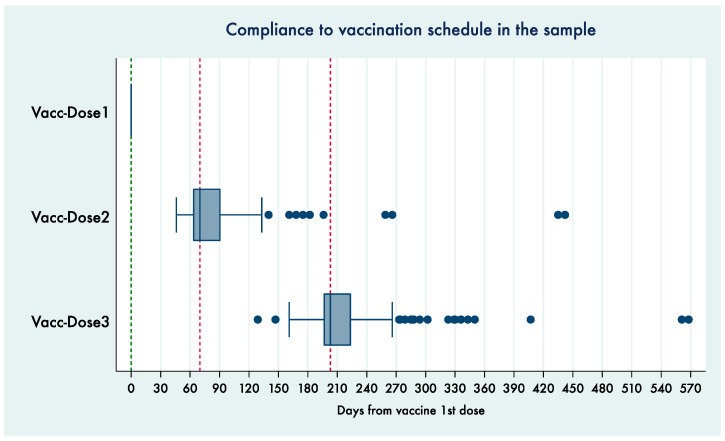
Box plot showing timings of second and third dose distributions.

A total of 512 sexually active women underwent the first 9vHPV dose administration during the time period considered for the study (age at first dose: median 30.5, 25th percentile 26, 75th percentile 36.5, min 14, max 70). Among them, 499 performed the second dose (at a median distance of 70 days—10 weeks—from the first dose) and 474 the third dose (at a median distance of 203 days—29 weeks—from the first dose). The distribution of dose timings since the date of the first dose is shown in Figure 1.

Dates and results of a total of 1006 Pap smears on 421 women and 718 HPV tests on 368 women were collected by the hospital information system platform of Fondazione Policlinico Gemelli or by asking the vaccinated women for the tests performed outside the Gemelli Health Care System.

Among the Pap smears, 364 were positive for ASC-US/L-SIL and 642 were negative. Regarding the timing of the collected tests, the oldest one was performed 2975 days (8.1 years) before the first dose administration, and the latest one was performed 1273 days (3.5 years) after the first dose administration. Half of the tests were performed at least 57 days before the first dose (median distance from the first dose), and 50% of the tests (IQR of distance from the first dose) were performed between 229 days before and 233 days after the first dose.

Among the HPV tests, 459 were positive for HPV-HR types, 53 showed HPV-LR types and 206 were totally negative. HPV 16 was the most frequent genotype detected in both single and co-infections. Regarding the timing of the collected tests, the oldest one was performed 3445 days (9.4 years) before the first dose administration, and the latest one was performed 1273 days (3.5 years) after the first dose administration. Half of the tests were performed at least 84 days before the first dose (median distance from the first dose), and 50% of the tests (IQR of distance from the first dose) were performed between 308 days before and 233 days after the first dose.

**Figure 2:**
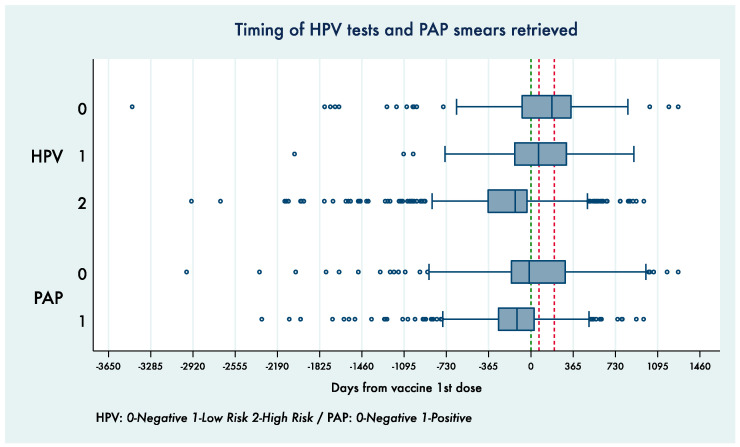
Time distribution for different HPV test and Pap smear results retrieved, where day 0 represents the vaccination 1st dose administration date for each enrolled woman.

The distribution of testing times, stratified for specific results, is shown in the box plots reported in Figure 2.

It can be noted that around 75% of the positive Pap and HPV-HR tests were performed before the vaccine first dose, whereas negative Pap or HPV-LR results were more frequently reported in tests performed after the first dose.

### 3.2. Time to Test Negativization

Since dates for Pap and HPV tests collected by the enrolled women ranged from years before the vaccine first dose to years after the administration of the last dose, and for many women more than one test of the same type was retrieved, we tried to analyze the data to understand if the exposure to vaccine doses could have reduced the time span between a positive test (Pap+ or HPV HR+) and a following negative test of the same type. Therefore, we divided the experience of each enrolled woman for which a Pap+ test (or an HPV HR+ test), followed by at least another Pap test (HPV test), regardless of the result, was retrieved into four time intervals: the pool of days from the date of a test performed before the first dose and the date in which the first dose was administered will become unexposed person-days, and each following negative test will be considered a spontaneous regression; all the time intervals after the first dose and the test negativization after the first, second and third doses may have been influenced (more or less) by the exposure to the vaccine. Numbers of women, cumulative person-days and negative tests following a previous positive test occurring during each individual time span in different vaccination statuses are summarized in Table 1.

#### 3.2.1. Time to PAP Smear Negativization

Among the 421 women with at least one Pap smear recorded, 207 (49.2%) reported at least one positive Pap smear followed by another Pap smear. A total of 191 women had at least 1 day of follow-up after a positive Pap smear before the first dose (47,027 person-days in total, with 65 negative tests), 124 had at least 1 day of follow-up between the first and the second dose (9062 person-days in total, with 11 negative tests), 115 had at least 1 day of follow-up between the second and the third dose (13,266 person-days in total, with 26 negative tests) and 93 had at least 1 day of follow-up after the third dose (14,254 person-days in total, with 72 negative tests).

**Figure 3:**
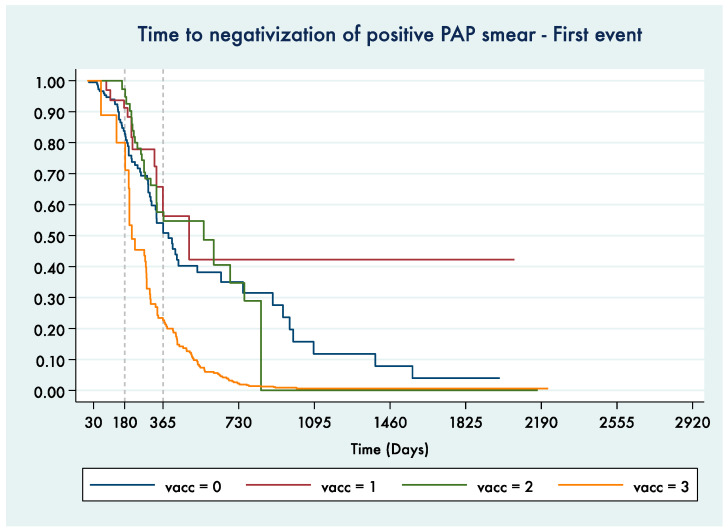
Kaplan–Meier curve for time to negativization of a positive Pap smear. Only the time to the 1st negative Pap smear after a positive Pap smear was considered (0—before vaccination, 1—exposed to 1st dose, 2—exposed to 1st and 2nd dose, 3—exposed to all 3 doses).

Figure 3 shows the Kaplan–Meier curve for the time to Pap smear+ negativization comparing time periods referring to women in different vaccinal statuses.

The log-rank test showed high statistical significance for the difference among the vaccinal status groups (*p* < 0.0001). After verification performed by univariate and multivariate Cox regression models, no confounding by age at the test date or vaccine administration date or by conization treatment performed within a year before the first dose (37/207 women were treated) was identified (Supplementary Material Tables S1 and S2).

#### 3.2.2. Time to HPV HR+ Negativization

Among the 368 women with at least one HPV test recorded, 198 (53.8%) reported at least one HPV-HR+ test followed by another HPV test. A total of 194 women had at least 1 day of follow-up after a positive HPV HR+ test before the first dose (88,814 person/days in total, with 23 negative tests), 151 had at least 1 day of follow-up between the first and the second dose (11,527 person/days in total, with 4 negative tests), 147 had at least 1 day of follow-up between the second and the third dose (19,377 person/days in total, with 13 negative tests) and 126 had at least 1 day of follow-up after the third dose (24,469 person/days in total, with 77 negative tests).

**Figure 4:**
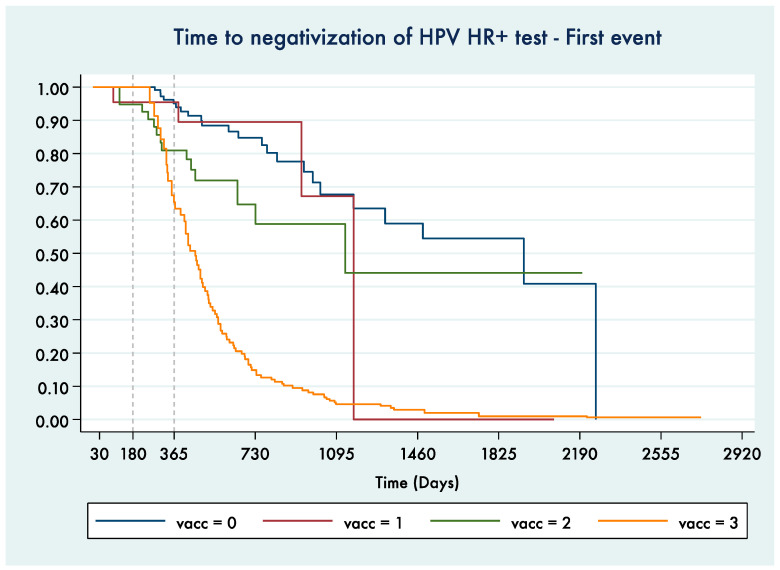
Kaplan–Meier curve for time to negativization of an HPV HR+ test. Only the time to the 1st HPV LR+ or HPV negative test after a positive HPV HR+ test was considered (0—before vaccination, 1—exposed to 1st dose, 2—exposed to 1st and 2nd dose, 3—exposed to all 3 doses).

Figure 4 shows the Kaplan–Meier curve for time to HPV-HR+ test negativization (to HPV-LR or totally negative) comparing time periods referring to women in different vaccinal statuses.

The log-rank test showed, also in this case, high statistical significance for the difference among the vaccinal status groups (*p* < 0.0001). After verification performed by univariate and multivariate Cox regression models, no confounding by age at the test date or vaccine administration date or by conization treatment performed within a year before the first dose (46/198 women were treated) was identified (Supplementary Material Tables S3 and S4).

#### 3.2.3. Comparing HRs for Time to Negativization for Pap and HPV HR+ Tests

Since no confounding effect by age at event or treatment performed within a year before the first dose was shown in multivariate Cox regression models, Table 2 shows a comparison of the results of the univariate Cox regression models for the time to negativization of Pap and HPV HR+ tests.

Vaccination doses show a stronger effect in shortening the negativization time for both HPV HR+ tests and positive Pap smears after schedule completion, and already after the second dose for the HPV HR+ test.

### 3.3. Safety

Finally, we can point out the good tolerability of the vaccine in our cohort. We found no serious adverse effects (AEs), and AEs were mainly local reactions (limited to pain and swelling at the injection site) in 50 women (9.8% of the sample). The only event of clinical relevance was one episode of supra-clavicular reactive lympho-adenopathy homolateral to the injection site after the first dose in a 25-year-old, who fully recovered in 15 days after the injection.

## 4. Discussion

The rationale for vaccinating adult women comes from the observation that the incidence of HPV infections decreases with age but it remains prominent even years after sexual debut, and that the incidence of HPV infection shows a second peak around 45–50 years [30]. Furthermore, many studies report that the level of protection conferred by natural infection is variable, as HPV HR types show numerous immunological escape mechanisms, and only 50% of infected women produce circulating antibodies [38].

Thus, although viral persistence appears to be the predominant cause of HPV infection in adult women, new infections may occur, and it is impossible to discriminate whether a new infection represents a new acquisition or a reactivation of a latent infection. Even if the vaccine therapeutic properties on pre-existing HPV-related lesions are still doubtful, women already infected with an HPV genotype included in a vaccine may indeed benefit from protection against other HPV genotypes that are not part of the infection [39]. Moreover, data on the immunogenicity and safety of the 9vHPV vaccine in women up to 27–45 years of age have recently been published [40].

With this background, although the vaccine has maximum effectiveness if administered before exposure to the virus [24], and it has no recognized therapeutic purposes, even sexually active women can benefit from anti-HPV immunization.

Previous diagnosis and treatment of an HPV-related lesion did not represent a contraindication to vaccination. Long-term observational cohort studies of vaccinated populations [41,42] and recent retrospective and prospective clinical studies showed that the HPV vaccine (2-valent and 4-valent), even in patients already treated for HPV-related lesions of the uterine cervix (e.g., conizations, laser therapies) and/or of the lower genital tract (e.g., medical or surgical therapies for warts, VIN, VaIN), has an important role in reducing the recurrence of the lesions [43,44,45]. 

The aim of our retrospective, monocentric study was to evaluate in a time-to-event analysis the possible relationship among the vaccination status and the time to negativization of the commonly used diagnostics tests for HPV infection (Pap smears, HPV DNA tests) in sexually active women who underwent opportunistic 9vHPV vaccination while reporting a positive HPV DNA HR test or showing minor cytological abnormalities.

Our study deals with a population of women who, according to the clinical practice, will normally undergo a follow-up in view of a possible spontaneous regression of HPV infection, with or without cytological low-grade lesions. We observed a good tolerability and a good compliance, with many subjects completing the whole vaccination schedule in the regular time, even if it has to be considered that we are performing an “on-demand” vaccination with a copayment modality, and therefore the participants are generally very highly motivated. Our time-to-event curves for Pap smear and HPV-HR+ test negativization, comparing time periods referring to women in different vaccinal statuses, both show high statistically significant differences among the vaccinal status groups (*p* < 0.0001), with a suggestion of a stronger effect on HPV HR+ than on Pap smears. Neither age at vaccination/testing time nor having been treated for cervical lesions in the year before the first dose played any role as confounders or interaction variables.

To our knowledge, as, to date, there are no similar data reported in the literature about the follow-up of commonly used diagnostics tests for HPV infection results in the adult population after 9vHPV vaccination, our results may be of great interest in understanding a possible beneficial role of HPV vaccines in reducing the time for HPV clearance where an HPV infection pre-existed before the first dose. 

There are few data in the literature on the benefit of HPV vaccination in adult populations with an active HPV infection. The retrospective study of Garolla et al. [46] in male subjects (aged 25 to 40 years) of infertile couples with HPV semen infection showed that HPV detection on sperm was predictive of a negative pregnancy outcome. Adjuvant HPV vaccination with the 4-valent vaccine was associated with enhanced HPV healing in seminal cells and increased the rate of natural pregnancies and live births.

The study of Ferris et al. [47] on the prevalence, incidence and natural history of HPV infection in adult women aged 24–45 participating in a vaccine trial with 4-valent HPV suggested that women who are older than the age typically targeted by HPV vaccination programs are at risk for incident and persistent HPV anogenital infections, depending on sexual behavior. As mid-adult women acquire new HPV infections, administration of the 9vHPV vaccine could reduce HPV-related morbidity and mortality in this population [47]. A focus on adult women is therefore relevant to individuals who were never vaccinated against HPV, as well as those who remain susceptible to the 9vHPV types that are not covered in the 2-valent HPV or 4-valent HPV vaccines.

A recent pilot observational study, conducted in Greece [48], investigated the hypothesis that 2-valent or 4-valent HPV vaccination in adult women with low-grade cytology could prospectively alter HPV-related biomarkers. HPV vaccination (performed in 152/309 women enrolled) significantly reduced HPV DNA positivity rates for genotypes 16, 18 and 31 in women who tested DNA-positive for HPV16, 18 and 31 genotypes prior to vaccination, demonstrating an earlier clearance of HPV infection in comparison with the non-vaccinated women.

A plausible explanation of the biological mechanism underlying our results may be related to the prevention of the reinfection and reactivation of a latent infection or to the reduction in viral shedding, as advised by some immunological studies [26]. These immunological mechanisms have also been proposed to explain adjuvant vaccination effects in reducing the incidence of recurrent disease after surgical treatment of cervical and vulvar HSIL, as suggested by Ghelardi et al. [26,45]. 

When, in natural infection, the immune system happens to be ineffective in providing a long-lasting protection, HPV vaccination may prevent the loss of immunological effectiveness: in fact, the high antibody levels following vaccination seem to prevent new areas of epithelial infection, whether due to dissemination from existing sites of HPV infection or from new HPV exposure, and thus prevent disease. 

The importance of HPV vaccination of previously HPV-infected women is also substantiated in a recent publication by Vorsters et al., who consider this practice safe and to generate a high-level immune response [49]. The authors contended that even in women with a productive infection, such as in women with low-grade cytology, vaccination will lead to a potentially neutralizing amount of transudated anti-HPV antibodies in their cervico-vaginal secretions; therefore, vaccination could prevent infectious virion dissemination from existing sites of HPV infection. Moreover, it may be suggested that local alterations of the vaginal microenvironment, mediated by the increased levels of pro-inflammatory cytokines see in persistent HPV infection, will be modified, in vaccinated women, by the high levels of anti-HPV antibodies in the cervico-vaginal secretion, favoring an earlier clearance of HPV infection.

The main methodological limitations of this study are linked to the observational retrospective design, the need to rely on the results of Pap smears and HPV tests spontaneously performed at highly variable time intervals in different laboratories and the lack of data on potentially relevant risk factors for the history of HPV infections, such as sexual habits, smoking status and use of estro-progestins. In particular, regarding cytology and HPV testing interval times, it is worthy of note that while, in Italy, the national cervical screening program offers free cytology every 3 years in women aged 25–29 years and, from 2018, according to European guidelines [50], high-risk HPV DNA testing in women aged 30–65 years every 5 years, “opportunistic” screening performed at shorter time intervals by private gynecologists in sexually active patients is quite common [51]. Moreover, Pap smear and HPV test results were seldom synchronous, since in Italy, co-testing is not usually performed on the same sample, and this may have added further variability to the time intervals in test sequences. Finally, regarding the natural history of HPV infection across the lifespan, many occurrences (60–80% within 2 years) of spontaneous regression of low-grade cytological abnormality have been reported in the literature [28,29]. Therefore, it is also possible that our result of HPV HR+ and positive Pap smear negativizations can be due, at least partially, also to the natural course of HPV infection and not only to the exposure to vaccines doses. 

On the other hand, this study addresses IARC’s call for data on the natural history of HPV infection among older women [52], may help to better understand the natural history of HPV infection across the lifespan and the role of viral latency and can suggest further benefits of HPV vaccination strategies that target multiple age cohorts and achieve high vaccine uptake [53]. 

## 5. Conclusions

HPV disease prevention guidelines can be informed and improved by data on the prevalence, incidence and persistence of HPV infections at any age, and particularly among older women, who are typically not targeted for HPV immunization. 

Our study suggests that 9vHPV vaccination may have a promising role in shortening the clearance time of HPV HR+ or Pap smear positivity in sexually active adult women. Further prospective studies may confirm this mechanism which may help in boosting the prophylactic role of HPV vaccination in women older than 26 years of age. Extending HPV vaccination to this population has the potential to be of utmost importance both for current public health policy and for the long-term reduction in HPV-related cancers. 

## Figures and Tables

**Table 1:** Summary of data available for the different exposure times.

		Vaccine Status	With at Least One Pap Test	With at Least One PAP+ and One FU Pap Test			With at Least One HPV Test	With at Least One HPV HR+ Test and One FU HPV Test		
		Women	Women	Women	Person-Days	Negative Tests	Women	Women	Person-Days	Negative Tests
**Enrolled in the study**		512	421	207	83,609		368	198	144,187	
**Time intervals groups**										
**vacc = 0**	Unexposed to vaccine (control)	512		191	47,027	65		194	88,814	23
**vacc = 1**	Exposed to 1st dose	512		124	9062	11		151	11,527	4
**vacc = 2**	Exposed to 1st and 2nd dose	499		115	13,266	26		147	19,377	13
**vacc = 3**	Exposed to 1st, 2nd and 3rd dose	474		93	14,254	72		126	24,469	77

**Table 2:** Comparison of univariate Cox regression model results for positive Pap smear versus HPV HR+ negativization.

Vaccinal Status	Pap Smear + -> Pap Smear -	HPV HR+ -> HPV LR+, HPV−
No vaccine	1.00	1.00
1° dose	0.70 (0.36–1.33)	1.35 (0.47–3.93)
1° + 2° dose	0.95 (0.60–1.52)	2.07 (1.04–4.11) *
1° + 2° + 3° dose	2.66 (1.83–3.86) **	7.80 (4.83–12.60) **

Results shown as HR (95% C.I.), * *p* < 0.05, ** *p* < 0.0001.

## Data Availability

Data may be available upon request to the corresponding authors.

## Supplementary Materials

The following are available online at https://www.mdpi.com/article/10.3390/vaccines9060643/s1, Table S1: Comparison of Cox regression model results for the evaluation of a possible confounding effect of age at event for time to negativization of a positive Pap smear. Model 1 is the univariate model, Model 2 is the model including age as a continuous variable and Model 3 is the model including age in 5 classes; Table S2: Comparison of Cox regression model results for the evaluation of a possible confounding effect of treatment in the year before the 1st dose for time to negativization of a positive Pap smear. Model 1 is the univariate model, and Model 4 is the model including the history of a conization treatment in the year before the 1st dose; Table S3: Comparison of Cox regression model results for the evaluation of a possible confounding effect of age at event for time to negativization of an HPV HR+ test. Model 1 is the univariate model, Model 2 is the model including age as a continuous variable and Model 3 is the model including age in 5 classes; Table S4: Comparison of Cox regression model results for the evaluation of a possible confounding effect of treatment in the year before the 1st dose for time to negativization of an HPV HR+ test. Model 1 is the univariate model, and Model 4 is the model including the history of a conization treatment in the year before the 1st dose.
