# Supplementary material for: HPV and Cytology Testing in Women Undergoing 9-Valent HPV Opportunistic Vaccination: A Single-Cohort Follow Up Study
Source: Vaccines (Basel). 2021 Jun 12;9(6):643. doi: 10.3390/vaccines9060643 (PMC8231148; doi:10.3390/vaccines9060643)
Supplement: Supplementary file 1 [file vaccines-09-00643-s001.zip › vaccines-1219557-supplementary.pdf]

## Supplementary Materials

**Table S1.** Comparison of Cox regression model results for the evaluation of a possible confounding effect of age at event for time to negativization of a positive Pap smear. Model 1 is the univariate model, Model 2 is the model including age as a continuous variable and Model 3 is the model including age in 5 classes.

| Variable | Model 1 | Model 2 | Model 3 |
|---|---|---|---|
| *HPV Vaccine* | | | |
| No vaccine | 1.00 | 1.00 | 1.00 |
| 1° dose | 0.70 (0.36-1.33) | 0.69 (0.36-1.32) | 0.69 (0.36-1.32) |
| 1°+2° dose | 0.95 (0.60-1.52) | 0.94 (0.59-1.50) | 0.94 (0.59-1.51) |
| 1°+2°+3° dose | 2.66 (1.83-3.86) ** | 2.60 (1.79-3.78) ** | 2.58 (1.77-3.77) ** |
| *Age at event* | | | |
| +1 yr | | 0.99 (0.97-1.00) | |
| <25 | | | 1.00 |
| 25-30 | | | 1.18 (0.71-1.99) |
| 30-35 | | | 0.86 (0.49-1.51) |
| 35-45 | | | 1.01 (0.58-1.74) |
| >45 | | | 0.77 (0.37-1.59) |

- *Results shown as HR (95% C.I.), * p < 0.05, ** p < 0.0001.*

**Table S2.** Comparison of Cox regression model results for the evaluation of a possible confounding effect of treatment in the year before the 1st dose for time to negativization of a positive Pap smear. Model 1 is the univariate model, and Model 4 is the model including the history of a conization treatment in the year before the 1st dose.

| Variable | Model 1 | Model 4 |
|---|---|---|
| *HPV Vaccine* | | |
| No vaccine | 1.00 | 1.00 |
| 1° dose | 0.70 (0.36-1.33) | 0.70 (0.36-1.33) |
| 1°+2° dose | 0.95 (0.60-1.52) | 0.95 (0.60-1.52) |
| 1°+2°+3° dose | 2.66 (1.83-3.86) ** | 2.66 (1.84-3.87) ** |
| ***Women who underwent a conization within a year before the 1st dose*** | | |
| Not treated | | 1.00 |
| Underwent a conization within 365 days before 1st dose | | 1.05 (0.71-1.56) |

- *Results shown as HR (95% C.I.), * p < 0.05, ** p < 0.0001.*

**Table S3.** Comparison of Cox regression model results for the evaluation of a possible confounding effect of age at event for time to negativization of an HPV HR+ test. Model 1 is the univariate model, Model 2 is the model including age as a continuous variable and Model 3 is the model including age in 5 classes.

| Variable | Model 1 | Model 2 | Model 3 |
|---|---|---|---|
| *HPV Vaccine* | | | |
| No vaccine | 1.00 | 1.00 | 1.00 |
| 1° dose | 1.35 (0.47-3.93) | 1.34 (0.34-2.85) | 1.32 (0.45-3.84) |
| 1°+2° dose | 2.07 (1.04-4.11) * | 2.05 (1.03-4.09) * | 2.01 (1.01-4.01) * |
| 1°+2°+3° dose | 7.80 (4.83-12.60) ** | 7.78 (4.82-12.58) ** | 7.56 (5.22-13.67) ** |

| | | |
|---|---|---|
| *Age at event* | | |
| +1 yr | 0.99 (0.97-1.02) | |
| | | |
| <25 | | 1.00 |
| 25-30 | | 1.92 (0.67-5.50) |
| 30-35 | | 1.90 (0.66-5.49) |
| 35-45 | | 2.01 (0.71-5.73) |
| >45 | | 1.09 (0.32-3.72) |

- *Results shown as HR (95% C.I.), * p < 0.05, ** p < 0.0001.*

**Table S4.** Comparison of Cox regression model results for the evaluation of a possible confounding effect of treatment in the year before the 1st dose for time to negativization of an HPV HR+ test. Model 1 is the univariate model, and Model 4 is the model including the history of a conization treatment in the year before the 1st dose.

| Variable | Model 1 | Model 4 |
|---|---|---|
| *HPV Vaccine* | | |
| No vaccine | 1.00 | 1.00 |
| 1° dose | 1.35 (0.47-3.93) | 1.32 (0.45-3.84) |
| 1°+2° dose | 2.07 (1.04-4.11) * | 2.02 (1.01-4.02) * |
| 1°+2°+3° dose | 7.80 (4.83-12.60) ** | 7.65 (4.72-12.38) ** |
| | | |
| ***Women who underwent a conization within a year before the 1st dose*** | | |
| Not treated | | 1.00 |
| Underwent a conization within 365 days before 1st dose | | 1.28 (0.84-1.93) |

- *Results shown as HR (95% C.I.), * p < 0.05, ** p < 0.0001.*
